# Investigation of the Relationship Between Intraoperative Transcranial Motor Evoked Potential and Compound Muscle Action Potential in Corrective Spinal Deformity Surgery

**Published:** 2026-07-29

**Authors:** GO SATO, HIDETOSHI NOJIRI, HIROMITSU TAKANO, HIROSHI MUKAIDA, MUNEAKI ISHIJIMA

**Affiliations:** 1 Department of Orthopaedic and Motor Organ Medicine, Graduate School of Medicine, Juntendo University, Tokyo, Japan; 1 Department of Orthopaedic and Motor Organ Medicine, Graduate School of Medicine, Juntendo University, Tokyo, Japan; 2 Department of Orthopaedic Surgery, Faculty of Medicine, Juntendo University, Tokyo, Japan; 2 Department of Orthopaedic Surgery, Faculty of Medicine, Juntendo University, Tokyo, Japan; 3 Department of Clinical Engineering, Juntendo University Hospital, Tokyo, Japan; 3 Department of Clinical Engineering, Juntendo University Hospital, Tokyo, Japan; 4 Department of Clinical Engineering, Faculty of Health Science, Juntendo University, Tokyo, Japan; 4 Department of Clinical Engineering, Faculty of Health Science, Juntendo University, Tokyo, Japan

**Keywords:** transcranial motor evoked potential, compound muscle action potential, intraoperative monitoring, anesthetic fade, spinal deformity correction

## Abstract

**Objective:**

This study aimed to evaluate the relationship between transcranial motor-evoked potentials (Tc-MEPs) and compound muscle action potentials (CMAPs) during spinal deformity correction surgery and to assess the clinical utility of their simultaneous evaluation.

**Methods:**

We retrospectively analyzed 42 patients who underwent spinal deformity correction surgery and had reliable Tc-MEP and CMAP recordings available for correlation analysis. CMAPs were recorded from the median nerve after supramaximal stimulation, whereas Tc-MEPs were recorded from six target muscles. Correlation analyses of percentage changes in Tc-MEP and CMAP amplitudes were performed across three phase comparisons using four intraoperative measurement time points.

**Results:**

Significant positive correlations between CMAP and Tc-MEP amplitudes were observed mainly in the abductor pollicis brevis and abductor hallucis muscles at multiple surgical phases, whereas no consistent correlation was observed in the tibialis anterior muscle. Overall, these correlations were small to moderate. In three patients, Tc-MEP amplitude decreased by ≥ 70% during scoliosis correction. In one case, CMAP amplitude remained preserved despite marked Tc-MEP reduction, whereas in the other two cases, both Tc-MEP and CMAP amplitudes decreased simultaneously. All patients recovered after intraoperative intervention, and no postoperative neurological deficits were observed. These findings suggest that simultaneous evaluation of Tc-MEP and CMAP may provide complementary information for interpreting intraoperative waveform changes.

**Conclusions:**

Small-to-moderate correlations were observed between Tc-MEP and CMAP amplitudes during spinal deformity correction surgery. Although the strength of these correlations was limited, simultaneous evaluation of Tc-MEP and CMAP may aid interpretation of intraoperative Tc-MEP amplitude reductions and may represent a practical strategy for improving intraoperative decision-making.

## Introduction

Transcranial motor-evoked potentials (Tc-MEPs) are widely used in intraoperative neurophysiological monitoring to detect possible motor pathway injury at an early stage and thereby reducing the risk of postoperative neurological deficits. Tc-MEP monitoring is particularly important during spinal deformity correction surgery, in which the spinal cord is exposed to substantial mechanical stress and ischemic risk during corrective maneuvers. In recent years, advances in stimulation techniques and the use of anesthetic agents with less suppressive effects on Tc-MEPs have improved the feasibility and success rate of monitoring. However, Tc-MEP amplitudes are influenced by multiple factors, including spinal cord ischemia, anesthetic agents, neuromuscular blockade, muscle ischemia, and body temperature^1)^. Consequently, intraoperative reductions in Tc-MEP amplitude do not always directly indicate spinal cord injury and may complicate interpretation.

In addition, the phenomenon of “anesthetic fade,” defined as a gradual decline in Tc-MEP amplitude during prolonged surgery under propofol-based total intravenous anesthesia in the absence of overt neurological injury, has received increasing attention^2-4)^. This issue is particularly relevant in spinal deformity correction surgery, which is often prolonged and associated with substantial physiological and anesthetic influences on evoked potentials.

Because spinal deformity correction surgery often involves a long operative duration, substantial corrective manipulation, and complex anesthetic management, intraoperative Tc-MEP amplitude reductions may occur in the absence of neurological injury. Therefore, when Tc-MEP amplitude decreases are observed during these procedures, distinguishing true spinal cord compromise from changes caused by systemic or anesthetic-related factors is clinically important.

Compound muscle action potentials (CMAPs), obtained by direct peripheral nerve stimulation, have been used as an indicator of neuromuscular and muscle responsiveness^5-7)^. Previous studies by Tanaka et al. proposed CMAP normalization as a method to compensate for anesthetic fade-related changes in Tc-MEP amplitudes during prolonged propofol-based anesthesia^5-7)^. Because CMAPs reflect peripheral motor nerve conduction, neuromuscular junction transmission, and muscle response distal to the central motor pathways, simultaneous evaluation of Tc-MEP and CMAP may provide complementary information when intraoperative Tc-MEP amplitude reductions are observed. In particular, when both Tc-MEP and CMAP amplitudes decrease simultaneously, systemic or anesthetic-related influences, including anesthetic fade or residual neuromuscular blockade, may be suspected. Conversely, a selective decrease in Tc-MEP amplitude with preserved CMAP may suggest a more central mechanism, including possible spinal cord compromise.

Therefore, in this study, we investigated the relationship between Tc-MEP and CMAP during spinal deformity correction surgery and evaluated the clinical utility of their simultaneous evaluation in intraoperative monitoring.

## Materials and Methods

### Subjects

This study enrolled 53 patients who underwent spinal deformity correction surgery at our spinal center between October 1, 2021, and May 31, 2023. Patients who underwent reoperation, had neuromuscular disorders, or were undergoing dialysis were excluded. Cases in which a reproducible CMAP waveform with a clear peak-to-peak amplitude could not be obtained after supramaximal stimulation were excluded from the correlation analysis.

### Anesthesia

All surgeries were performed under total intravenous anesthesia (TIVA) using propofol and opioids (fentanyl and remifentanil). Muscle relaxants were administered only during tracheal intubation. When Tc-MEP waveforms were not detectable during the initial intraoperative evaluation, 200 mg of sugammadex sodium was administered intravenously, and surgery proceeded only after reproducible Tc-MEP waveforms were confirmed.

In adult patients, anesthesia was induced using a target-controlled infusion (TCI) pump with a target propofol concentration of 3 μg/mL and maintained at 2-4 μg/mL, adjusted to maintain a bispectral index (BIS) value between 40 and 60. In patients aged 16 years or younger, anesthesia was induced with a bolus dose of propofol (2.0 mg/kg) and maintained with continuous infusion at 6-8 mg/kg/h.

Continuous arterial blood pressure monitoring was performed and mean arterial pressure (MAP) was generally maintained at ≥ 60 mmHg. Core body temperature was monitored using a bladder temperature probe and maintained at approximately 36.0−37.0°C with active warming devices. Arterial blood gas analysis and electrolyte monitoring (sodium, potassium, calcium, and glucose) were performed as needed, and abnormalities were corrected within clinically acceptable ranges.

### Tc-MEP and CMAP monitoring

**Figure 1:**
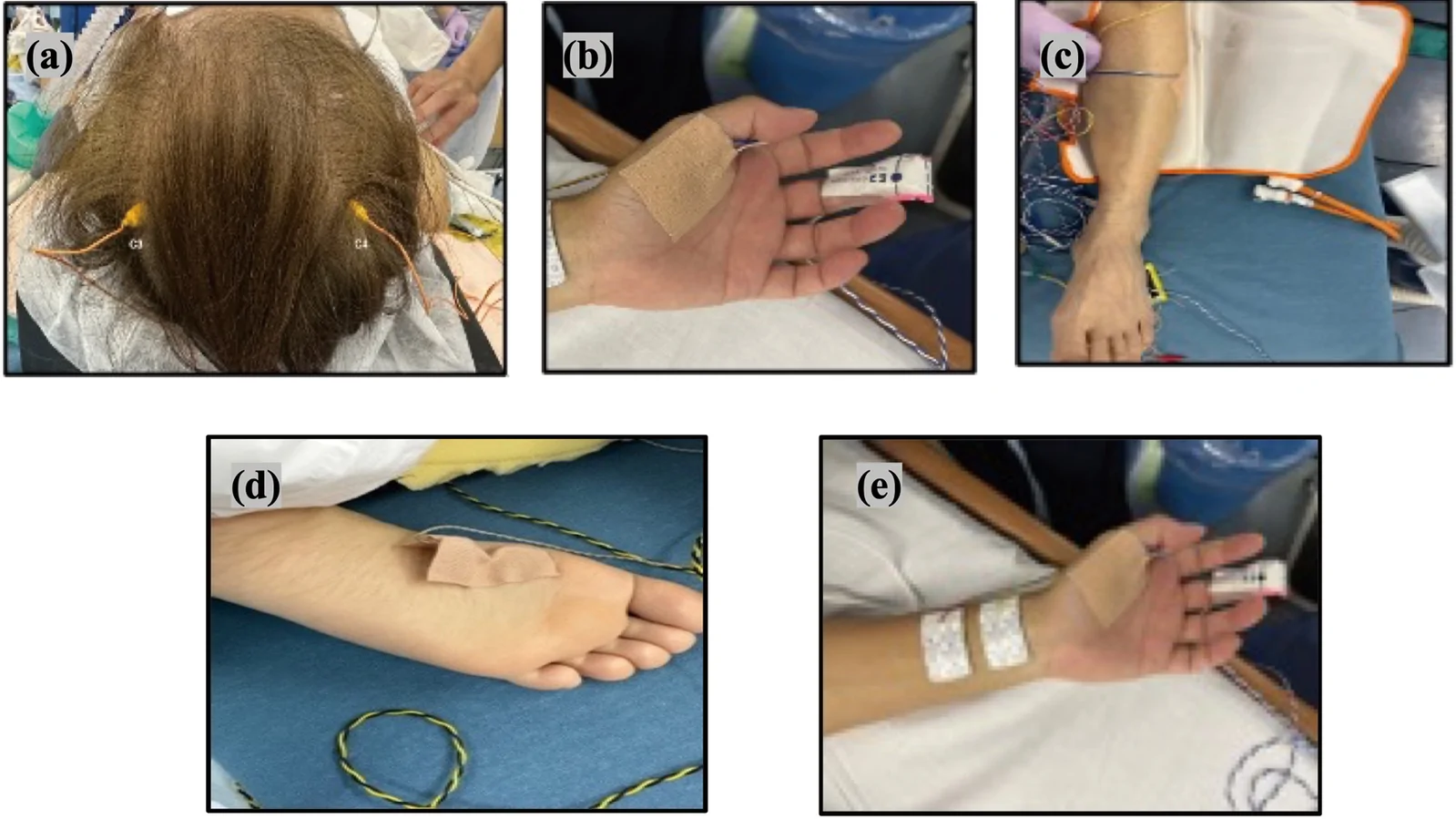
Setup for Tc-MEP and CMAP recording (a) Stimulation sites using corkscrew electrodes (DEM1001, Medtronic, Japan) placed at the C3 and C4 positions according to the International 10-20 System. (b) Recording site for the abductor pollicis brevis (APB). (c) Recording site for the tibialis anterior (TA). (d) Recording site for the abductor hallucis (AH). (e) CMAP recording setup using disposable surface electrodes (V-bit road electrodes) applied over the median nerve at the wrist.

Tc-MEPs were monitored using a neurostimulation device (Neuro Master MEE-2200, NIHON KOHDEN, Japan). Stimulation electrodes were placed on the scalp at the C3 and C4 positions in accordance with the international 10-20 electrode placement system using DEM1001 corkscrew electrodes (Medtronic, Tokyo, Japan) as stimulation sites (Figure 1). The stimulation intensity was set at a constant voltage between 200 V and 400 V, which was 20% above the stimulation threshold. A train of five pulses was delivered with a pulse duration of 0.5 ms and an interstimulus interval (ISI) of 2.0 ms.

Recordings were obtained using subcutaneous needle electrodes (PRASS; Medtronic, Japan) and disposable subdermal needle electrodes (NE-220B; NIHON KOHDEN, Japan) placed bilaterally on the abductor pollicis brevis (APB), tibialis anterior (TA), and abductor hallucis (AH) muscles (Figure 1).

CMAPs were recorded from the median nerve using disposable V-bit road electrodes (NIHON KOHDEN, Japan) with single-pulse electrical stimulation at the wrist level. The stimulation intensity was set between 20 and 50 mA and adjusted to achieve a supramaximal response, defined as the intensity yielding the maximal peak-to-peak amplitude (Figure 1). A reliable CMAP was defined as a reproducible waveform with a clear peak-to-peak amplitude obtained after supramaximal stimulation. CMAP recordings were influenced by individual anatomical factors, including body habitus and subcutaneous fat thickness. In some patients, reliable CMAP waveforms could not be obtained because venous lines or anatomical constraints interfered with accurate localization of the median nerve.

As a criterion for significant amplitude reduction, the Intraoperative Monitoring Working Group of the Japanese Society for Spine and Spinal Cord Surgery recommends a reduction of 30% of the baseline (peak-to-peak) amplitude, which may indicate potential neurological injury. In accordance with these guidelines, our institution defines the Tc-MEP warning threshold as a reduction of 70% or more from the baseline amplitude^8)^.

### Analysis

**Figure 2:**
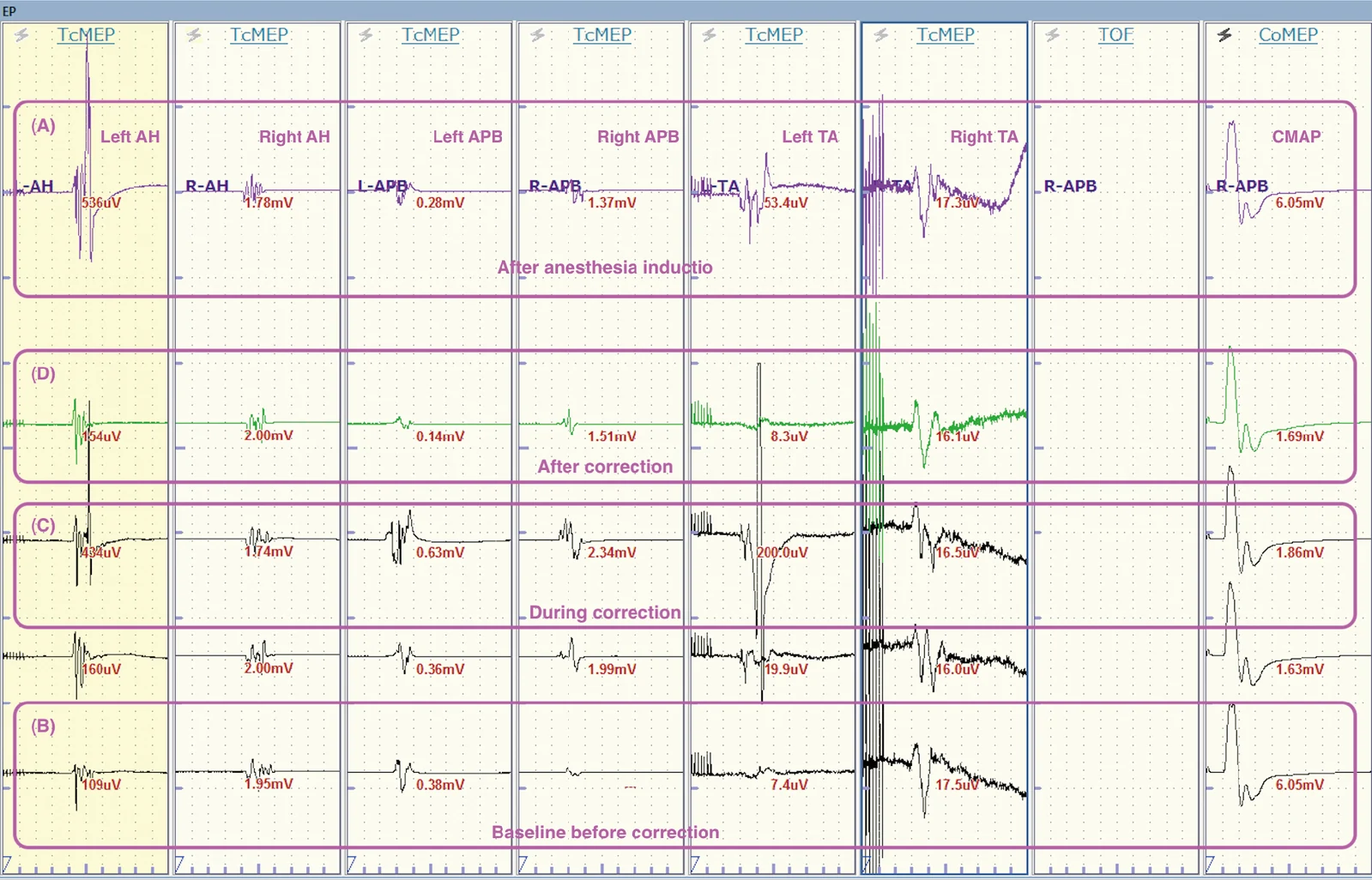
Representative intraoperative waveform changes at the four measurement time points: after anesthesia induction (A), baseline before scoliosis correction (B), during scoliosis correction (C), and after completion of scoliosis correction (D).

Measurements were obtained at four time points: (A) after anesthesia induction, (B) baseline before scoliosis correction, (C) during scoliosis correction, and (D) after completion of scoliosis correction (Figure 2). Percentage changes in Tc-MEP and CMAP amplitudes were calculated for each phase comparison. Correlation analyses were performed for three phase comparisons: A-B (after anesthesia induction and baseline), B-C (baseline and during correction), and B-D (baseline and after correction). Pearson correlation coefficients were calculated to evaluate the relationship between percentage changes in Tc-MEP and CMAP amplitudes for each monitored muscle. Scatter plots with fitted regression lines were used to visually examine the linear relationship between the two variables. All statistical analyses were performed using R software (version 4.5.2; R Foundation for Statistical Computing, Vienna, Austria). A two-sided p value < 0.05 was considered statistically significant.

## Results

### Study population

Among the 53 patients who underwent spinal deformity correction surgery during the study period, 42 cases with reliable Tc-MEP and CMAP recordings were included in the final analysis. In the remaining 11 patients, reliable CMAP waveforms could not be obtained because of difficulty in accurately localizing the median nerve, mainly owing to vascular lines or anatomical constraints. Among the 42 analyzed cases, three patients underwent osteotomy procedures (one Ponte osteotomy and two hemivertebra resections). Baseline demographic and surgical characteristics of the analyzed patients are summarized in Table 1.

**Table 1:** Baseline patient and surgical characteristics (n = 42)

Variable	Value
Age, years	29 (16-52)
Sex (female/male), n (%)	32 (76.2) / 10 (23.8)
BMI, kg/m^2^	19.5 (17.9-21.9)
ASA physical status, n (%)	
I-II	40 (95.2)
III	2 (4.8)
Etiology of deformity, n (%)	
Degenerative	31 (73.8)
Idiopathic	11 (26.2)
Main curve Cobb angle, °	46 (45-56)
Osteotomy performed, n (%)	3 (7.1)
Operative time, min	286 (218-368)
Estimated blood loss, mL	100−1000

Data are presented as median (interquartile range), number (percentage), or range for estimated blood loss. Preoperative Cobb angle was assessed in 39 patients without osteotomy.

### Correlation between percentage changes in Tc-MEP and CMAP amplitudes

Pearson correlation coefficients between percentage changes in Tc-MEP and CMAP amplitudes at different surgical phases are summarized in Table 2.

**Table 2:** Pearson correlation coefficients between percentage changes in Tc-MEP and CMAP amplitudes at different surgical phases

Phase	Left APB (r, p)	Right APB (r, p)	Left TA (r, p)	Right TA (r, p)	Left AH (r, p)	Right AH (r, p)
After anesthesia induction vs baseline (A-B)	**0.53, < 0.001**	**0.40, 0.008**	0.10, 0.58	0.21, 0.249	**0.54, < 0.001**	**0.36, 0.021**
Baseline vs during correction (B-C)	**0.42, 0.005**	**0.54, < 0.001**	0.26, 0.137	**0.40, 0.029**	**0.46, 0.002**	**0.35, 0.022**
Baseline vs after correction (B-D)	**0.48, 0.001**	**0.54, < 0.001**	0.28, 0.138	0.30, 0.086	**0.38, 0.012**	**0.53, < 0.001**

Bold values indicate statistically significant correlations (p < 0.05). A, after anesthesia induction; B, baseline before scoliosis correction; C, during scoliosis correction; D, after completion of scoliosis correction. A-B, percentage change from A to B; B-C, percentage change from B to C; B-D, percentage change from B to D. APB, abductor pollicis brevis; TA, tibialis anterior; AH, abductor hallucis; Tc-MEP, transcranial motor evoked potential; CMAP, compound muscle action potential.

In the comparison between after anesthesia induction and baseline (A-B), significant positive correlations were observed in the Left APB (r = 0.53, p < 0.001), Right APB (r = 0.40, p = 0.008), Left AH (r = 0.54, p < 0.001), and Right AH (r = 0.36, p = 0.021). No significant correlations were identified in the tibialis anterior muscles.

In the comparison between baseline and during correction (B-C), significant positive correlations were observed in the Left APB (r = 0.42, p = 0.005), Right APB (r = 0.54, p < 0.001), Right TA (r = 0.40, p = 0.029), Left AH (r = 0.46, p = 0.002), and Right AH (r = 0.35, p = 0.022). No significant correlation was observed in the Left TA (r = 0.26, p = 0.137).

In the comparison between baseline and after correction (B-D), significant positive correlations were observed in the Left APB (r = 0.48, p = 0.001), Right APB (r = 0.54, p < 0.001), Left AH (r = 0.38, p = 0.012), and Right AH (r = 0.53, p < 0.001), whereas no significant correlations were observed in either tibialis anterior muscle.

Overall, small-to-moderate positive correlations between Tc-MEP and CMAP amplitudes were observed mainly in the APB and AH muscles across the surgical phases.

### Representative scatter plots

**Figure 3:**
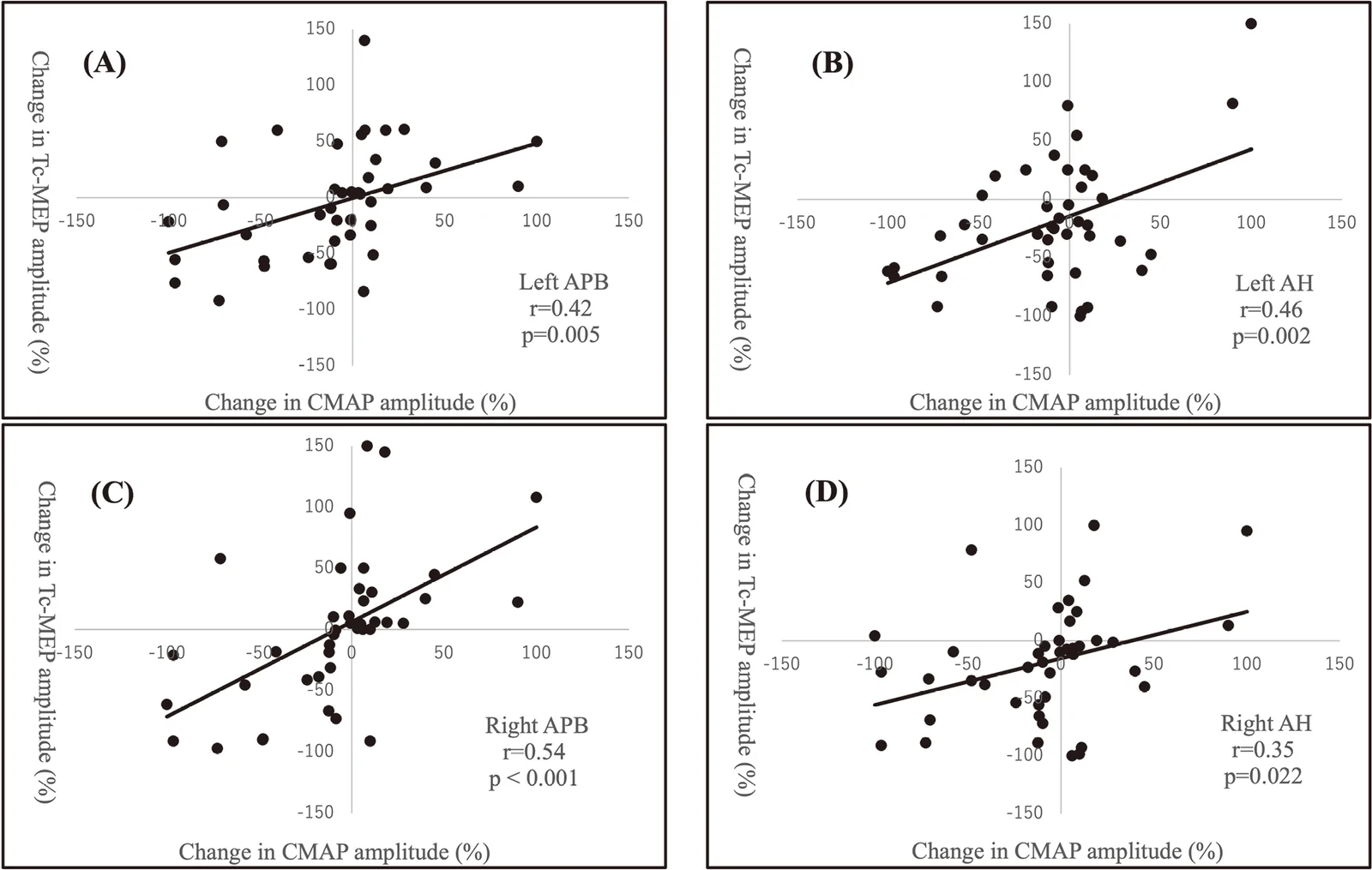
Correlation between Tc-MEP and CMAP amplitude changes during scoliosis correction Scatter plots illustrate the relationship between percentage changes in Tc-MEP and CMAP amplitudes from the baseline before scoliosis correction to the correction phase. (A) Left APB, (B) Left AH, (C) Right APB, and (D) Right AH. Pearson correlation coefficients (r) and corresponding p values are shown in each panel.

Representative scatter plots of significant correlations during the correction phase (B-C) are shown in Figure 3. Positive correlations were observed in the Left APB, Left AH, Right APB, and Right AH muscles, consistent with the overall trend shown in Table 2.

### Representative cases with marked Tc-MEP amplitude reduction

A decrease in Tc-MEP amplitude of ≥ 70%, including complete disappearance, was observed in three patients during scoliosis correction.

**Figure 4:**
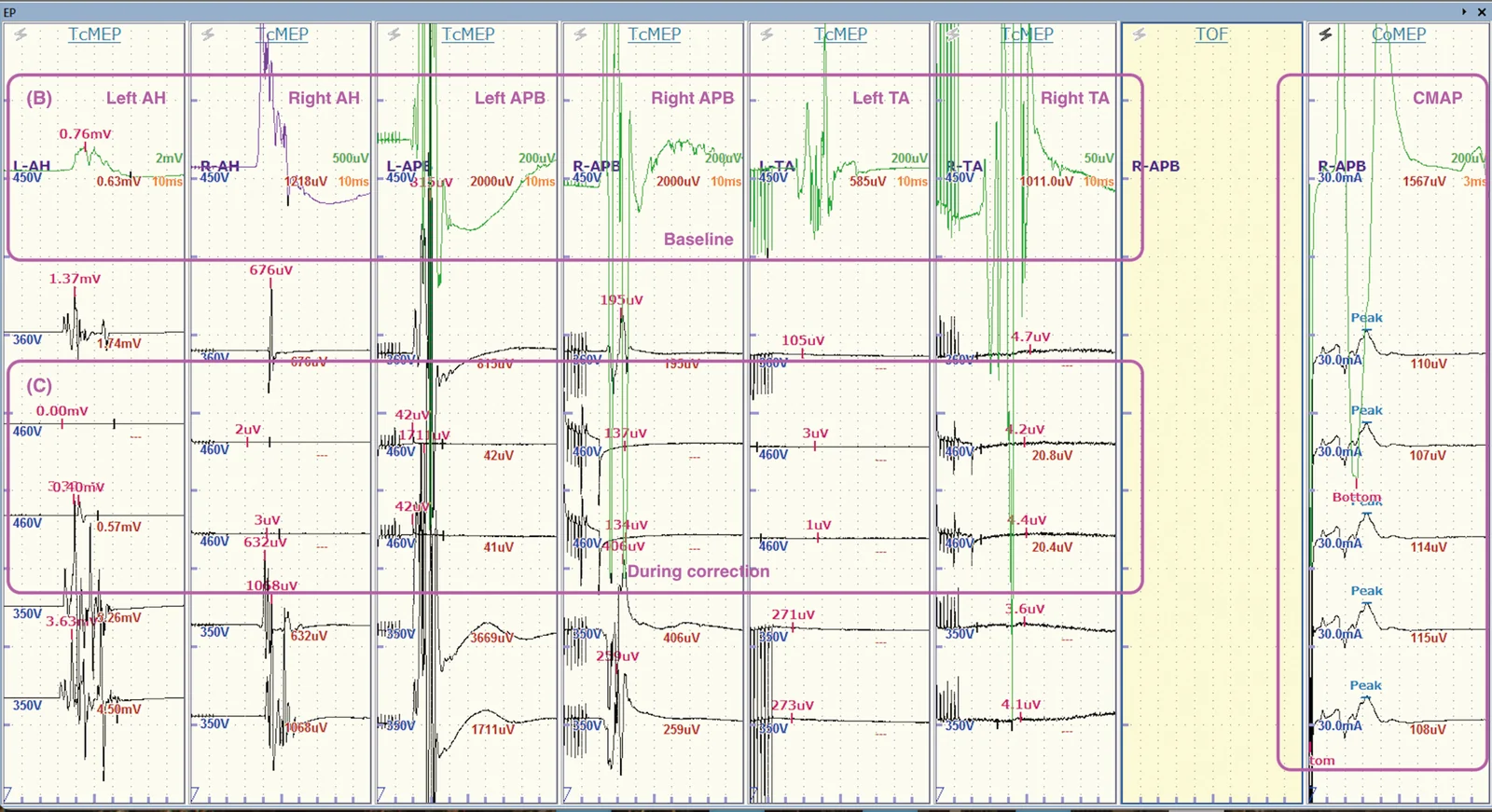
Representative intraoperative waveform changes in a case showing marked reduction in Tc-MEP amplitudes during correction while CMAP amplitude remained preserved. Waveforms are shown at baseline (B) and during correction (C).

In one case, Tc-MEP amplitudes markedly decreased while CMAP amplitude remained preserved (Figure 4), suggesting possible spinal cord stress rather than systemic anesthetic influence.

**Figure 5:**
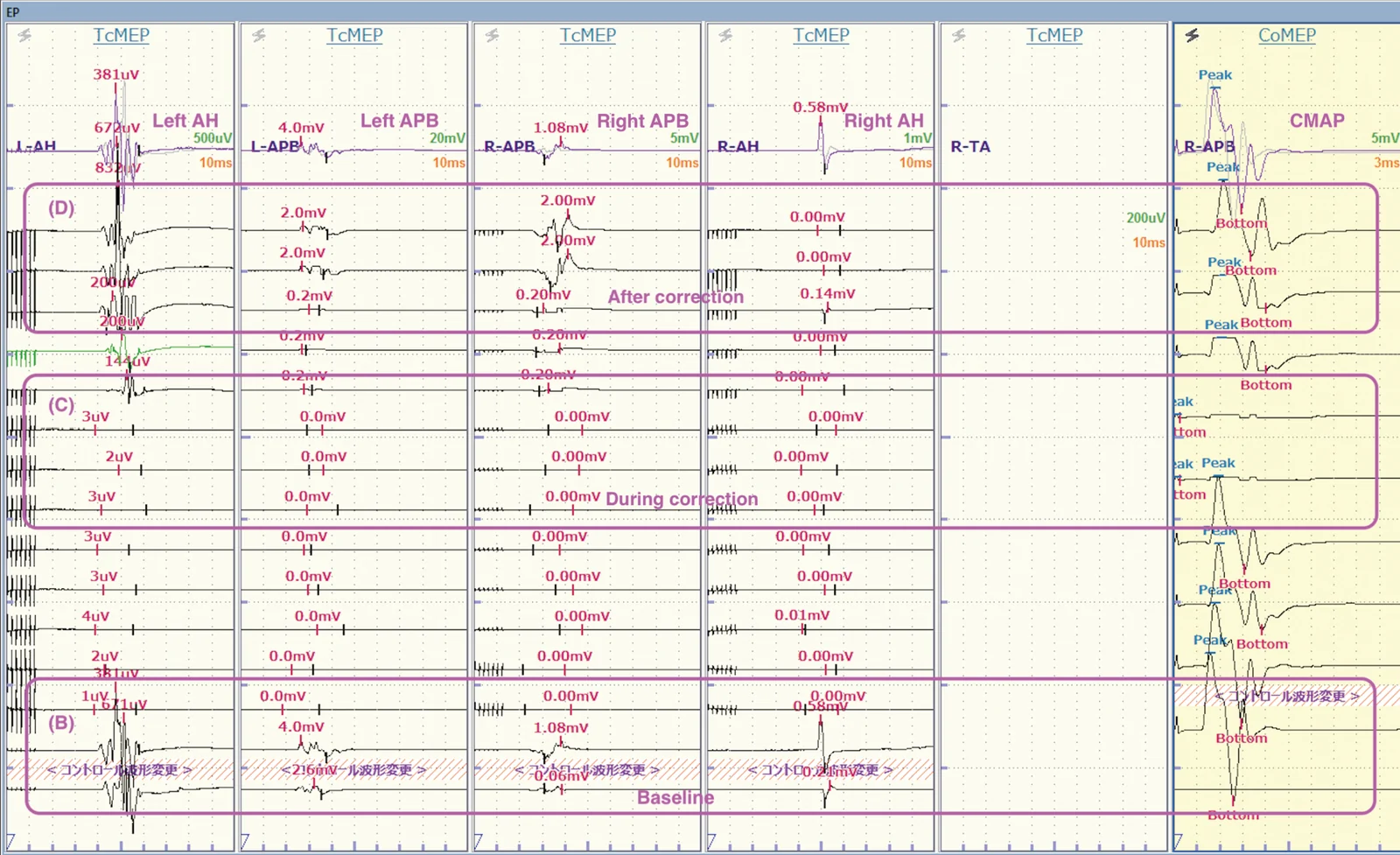
Representative intraoperative waveform changes in a case showing simultaneous reductions in Tc-MEP and CMAP amplitudes during correction. Waveforms are shown at baseline (B), during correction (C), and after correction (D). The tibialis anterior muscle was not monitored in this case. Waveforms are presented in the original intraoperative display order.

In the remaining two cases, simultaneous reductions were observed in both Tc-MEP and CMAP amplitudes (Figure 5), suggesting systemic or anesthetic-related influences, such as anesthetic depth or residual neuromuscular blockade.

In all three cases, Tc-MEP amplitudes recovered after intraoperative intervention, and no postoperative neurological deficits were observed. In addition, no patients without intraoperative warning findings developed new postoperative neurological deficits.

## Discussion

The present study demonstrated small-to-moderate correlations between Tc-MEP and CMAP amplitudes in several muscles during spinal deformity correction surgery. Simultaneous evaluation of these signals provided additional information for interpreting intraoperative Tc-MEP amplitude reductions.

Previous studies have shown that Tc-MEP signals are influenced by multiple systemic and anesthetic factors, including anesthetic depth, neuromuscular blockade, body temperature, and hemodynamic changes^3, 9, 10)^. Although CMAP monitoring has been proposed as a method to assess peripheral neuromuscular transmission, few studies have examined the relationship between Tc-MEP and CMAP amplitudes during spinal deformity correction surgery^5-7)^.

Intraoperative decreases in muscle-recorded Tc- MEP amplitudes may occur not only because of true spinal cord compromise but also because of systemic or anesthetic-related factors. In this study, anesthetic-related factors include the effects of anesthetic depth and residual neuromuscular blockade on neuromuscular junction transmission and muscle responses, whereas systemic factors include hemodynamic instability, body temperature changes, and oxygenation status. Because CMAPs are elicited by direct peripheral nerve stimulation and reflect peripheral motor nerve conduction, neuromuscular junction transmission, and muscle response distal to the central motor pathways, simultaneous CMAP monitoring may help determine whether a Tc-MEP decrease is accompanied by reduced peripheral neuromuscular responsiveness. Therefore, when both Tc- MEP and CMAP amplitudes decrease simultaneously, systemic or anesthetic-related influences, including anesthetic fade or residual neuromuscular blockade, should be considered. In contrast, a selective decrease in Tc-MEP amplitude with preserved CMAP may suggest a more central mechanism, including possible spinal cord compromise. However, CMAP monitoring does not directly exclude spinal cord injury but provides complementary information for interpreting Tc-MEP amplitude changes.

In the present study, correlation analyses revealed small-to-moderate correlations between CMAP and Tc-MEP amplitudes in several muscles, particularly in the APB and AH muscles. These findings reflect the multifactorial nature of Tc-MEP generation, which depends on both central motor pathways and peripheral neuromuscular transmission. Accordingly, the clinical value of CMAP lies not in mathematically correcting Tc-MEP amplitudes but in providing complementary information that may aid interpretation of waveform changes^5-7)^.

Representative waveform changes are illustrated in Figures 4 and 5. In one case, Tc-MEP amplitudes decreased by more than 70% during correction while CMAP amplitudes remained preserved, suggesting possible spinal cord stress rather than systemic or anesthetic-related influences. In contrast, another case demonstrated simultaneous reductions in both Tc-MEP and CMAP amplitudes during correction, followed by recovery after intraoperative management. These cases illustrate how simultaneous evaluation of Tc-MEP and CMAP signals may assist in distinguishing potential spinal cord compromise from systemic or anesthetic-related influences during scoliosis correction.

Although only a limited number of cases clearly demonstrated differentiation between correction- related and systemic or anesthetic-related causes of Tc-MEP reduction, the combined evaluation of Tc-MEP and CMAP signals may contribute to more accurate intraoperative interpretation of waveform changes. This approach may be particularly useful during prolonged spinal deformity surgery, where anesthetic fade and systemic physiological factors can influence Tc-MEP amplitudes^2-4)^.

**Figure 6:**
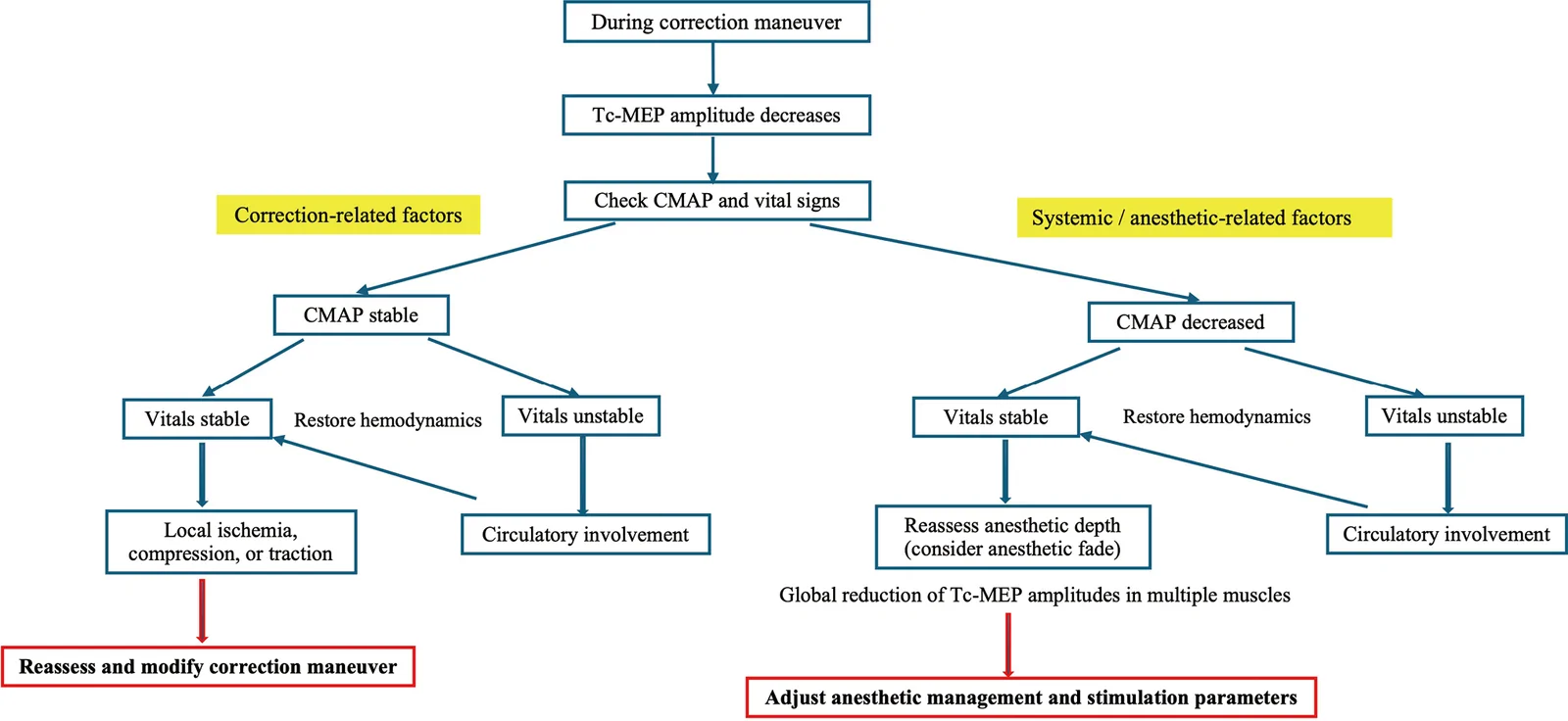
Flowchart of intraoperative evaluation for Tc-MEP amplitude reduction during scoliosis correction

Based on these observations, we developed a decision-making flowchart to facilitate rapid intraoperative interpretation of Tc-MEP amplitude reductions (Figure 6). When Tc-MEP amplitude decreases by ≥ 70% while CMAP amplitude remains preserved, spinal cord compromise should be considered, and reduction or temporary suspension of corrective maneuvers may be warranted. In contrast, when both Tc-MEP and CMAP amplitudes decrease simultaneously, systemic or anesthetic-related factors, such as anesthetic depth, hemodynamic instability, body temperature, or residual neuromuscular blockade, should first be evaluated and corrected before modifying the surgical strategy. These findings suggest that simultaneous evaluation may improve intraoperative decision-making when Tc-MEP waveform changes occur.

This study has several limitations. First, it was a retrospective single-center study with a relatively small sample size. Second, reliable CMAP recordings could not be obtained in all patients because of anatomical and procedural constraints. Third, multiple statistical comparisons were performed, which may increase the risk of type I error. Future prospective studies with standardized recording protocols are warranted to validate these findings. Taken together, our findings suggest that simultaneous monitoring of Tc-MEP and CMAP may provide a practical strategy for improving the intraoperative interpretation of Tc-MEP waveform changes during spinal deformity correction surgery.

## Conclusion

This study demonstrated small-to-moderate correlations between Tc-MEP and CMAP amplitudes during spinal deformity correction surgery. Although the strength of the correlations was limited, simultaneous evaluation of Tc-MEP and CMAP provided complementary information that may aid interpretation of intraoperative Tc-MEP amplitude reductions. These findings suggest that simultaneous evaluation of Tc-MEP and CMAP may represent a practical strategy for improving intraoperative interpretation during spinal deformity correction surgery.

## Author contributions

GS and HN contributed to the study conception and design. GS contributed to the data collection. GS and HM performed the data analysis and interpretation. GS drafted the manuscript. HN, HT, and MI revised the manuscript critically. All the authors have read and approved the final version of the manuscript.

## Conflicts of interest statement

The authors declare that there are no conflicts of interest.
